# See It Best: A Propensity-Matched Analysis of Ultrasound-Guided versus Blind Femoral Artery Puncture in Balloon-Expandable TAVI

**DOI:** 10.3390/jcm13051514

**Published:** 2024-03-06

**Authors:** Marco Gennari, Agnese Maccarana, Gaia Severgnini, Vittoria Iennaco, Alice Bonomi, Nicolò Capra, Federico De Marco, Manuela Muratori, Laura Fusini, Gianluca Polvani, Marco Agrifoglio

**Affiliations:** 1 Centro Cardiologico Monzino IRCCS, Department of Invasive Cardiology, Structural and Valvular Interventional Cardiology Unit, 20138 Milan, Italy; federico.demarco@cardiologicomonzino.it; 2 Centro Cardiologico Monzino IRCCS, Department of Cardiovascular Surgery, 20138 Milan, Italy; agnese.maccarana@unimi.it (A.M.); vittoria.iennaco@unimi.it (V.I.); gianluca.polvani@cardiologicomonzino.it (G.P.); marco.agrifoglio@cardiologicomonzino.it (M.A.); 3 Centro Cardiologico Monzino IRCCS, Department of Biostatistics, 20138 Milan, Italy; alice.bonomi@cardiologicomonzino.it (A.B.); nicolo.capra@cardiologicomonzino.it (N.C.); 4 Centro Cardiologico Monzino IRCCS, Department of Imaging, 20138 Milan, Italy; manuela.muratori@cardiologicomonzino.it (M.M.); laura.fusini@cardiologicomonzino.it (L.F.); 5 Department of Biomedical, Surgical and Dental Sciences, University of Milan, 20100 Milan, Italy

**Keywords:** TAVI, vascular complications, ultrasound, transfemoral

## Abstract

**Background**: Currently, transcatheter aortic valve implantation (TAVI) is the standard procedure recommended for patients over 75 years of age with symptomatic aortic valve stenosis. Percutaneous transfemoral (TF) access is the main route used to perform the procedure. Among periprocedural complications, access-related ones are the most frequent, potentially leading to prolonged in-hospital stays and transfusions. **Methods**: We performed a retrospective analysis of prospectively collected data on consecutive patients undergoing TF-TAVI with the latest generation balloon-expandable transcatheter valve between 2013 and 2022. **Results**: A total of 600 patients were analyzed, differentiating the population between ultrasound-guided and blind common femoral artery puncture. Valve Academic Research Consortium 3 (VARC-3)criteria were used to report at 30 days and follow-up. In our propensity-matched comparison of the two groups, we found a strong reduction in access-related complications in the echo-guided group, particularly in terms of reduction of major and minor bleedings. We also found a significant trend in reduction of local complications, such as pseudoaneurysms, hematomas, arterio-venous fistulas, dissection of the femoral or iliac arteries, and stenosis. **Conclusions**: Although there is a lack of consensus on the role of ultrasound-guided puncture, we found better outcomes for patients having an echo-guided puncture of the main access, particularly with regard to access-related complications, early mobilization, and early discharge home.

## 1. Introduction

As the clinical indications for transcatheter aortic valve implantation (TAVI) procedures have expanded from patients suffering from severe symptomatic aortic stenosis deemed to be at a high risk or too old for conventional surgery to a progressively larger population, further effort is needed to optimize outcomes and reduce complications.

Considering current guidelines on heart valve management [1], transfemoral (TF)-TAVI is the standard of treatment for patients over 75 years of age suffering from severe symptomatic aortic stenosis.

Among periprocedural complications, access-related complications carry significant morbidity and mortality, and despite the lower profile of latest-generation delivery catheters, their incidence is still high, reported in about 6–20% [2].

Access-related complications are linked to prolonged hospital stays, increased blood transfusions, and increased intra-hospital mortality.

Currently the transfemoral approach is the preferred access route, utilized in more than 90% of TAVI procedures. A percutaneous femoral vascular access is classically favored over surgical cut-down [3] because of better outcomes (i.e., faster healing, early deambulation, infection, and lymphorrhagia risk reduction) and a drop in in-hospital stays.

Percutaneous vascular access can be achieved via both traditional blind puncture, using anatomical landmarks (with or without fluoroscopy aid), or using an ultrasound-guided approach, which has gained more consensus in recent years.

In the most recent years, the use of the ultrasound-guided approach for femoral access has not only become established for arterial puncture but also for central venous cannulation. For example, it has been shown that real-time ultrasound-guided central subclavian vein cannulation has greater safety and quality and better first-time success compared with the blind anatomical landmark technique [4,5]. Similarly, other studies have observed better efficacy of the peripheral venous cannulation technique in the case of challenging intravenous access [6]. Poulsen et al. [7] showed that the use of ultrasound resulted in a three-fold increase in first-attempt success in patients with predicted challenging intravenous access compared to the traditional blind technique.

The main evidence for preferring ultrasound (US)-directed femoral puncture is derived from clinical registries and small clinical studies. In these settings, the systematic use of ultrasound-guided access has been shown to reduce access-related complications such as bleeding and vascular complications, as well as reducing the number of attempts and time to complete the procedure [8]. The aim of this report is to show our experience with blind versus US-guided femoral artery puncture for the TAVI procedure.

## 2. Materials and Methods

We have conducted a retrospective observational study on prospectively collected data, with patient follow-up over several years.

**Figure 1:**
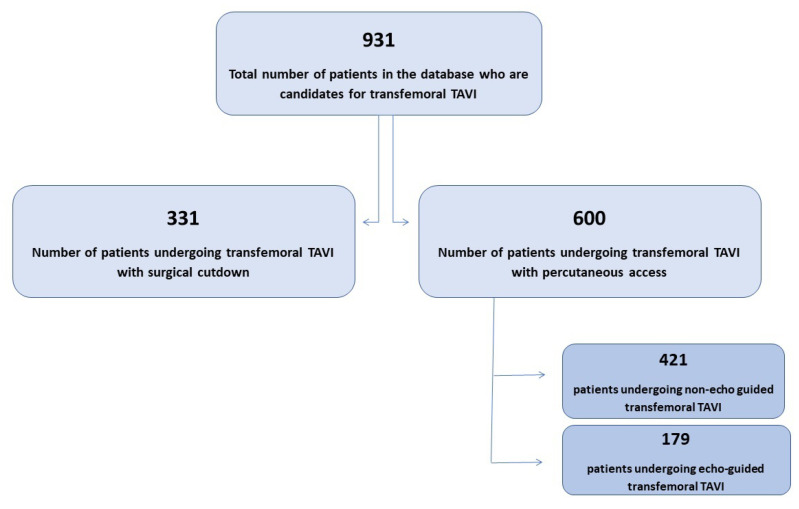
Flowchart of the study.

The study was carried out at the Centro Cardiologico Monzino (CCM) in Milan, Italy, from 2013 to 2022. A flowchart of the analysis is shown in Figure 1.

This study was conducted on 931 consecutive patients who underwent TF-TAVI. Of the overall population, 600 patients had a percutaneous access and were enrolled in the present analysis. This latter group was divided into two subgroups: group A (consisting of 421 patients) included patients having blind arterial puncture, and group B (consisting of 179 patients) had US-guided access.

All patients were approved for TF-TAVI by the local multidisciplinary team. A femoral artery caliber ≥ 5.5 mm was considered mandatory.

The SAPIEN 3 (S3) and SAPIEN 3 Ultra (S3U) (Edwards Lifesciences, Irvine, CA, USA) balloon-expandable transcatheter heart valves (BE-THV) were analyzed in this study, as they were the most frequent devices used at the time of this report.

We compared the perioperative outcomes and follow-up of US-guided and non-US-guided percutaneous transfemoral TAVI. We also compared the odds ratio of a peri-procedural complication between the two groups after adjusting for propensity score.

Follow-up was performed by phone calls, after obtaining verbal informed consent.

### 2.1. Ethical Committee

The number of the internal protocol approval is CCM1895, made by the “Comitato Etico territoriale Lombardia II”, Italy.

### 2.2. Echo-Guided Femoral Puncture Protocol

A standard linear vascular ultrasound echo probe is routinely used to identify the common femoral artery of the main access (and its bifurcation) in long-axis view. The echographic characteristics are also used to locate an adequate site of puncture on the common femoral artery, excluding segments with large branches and significant calcifications. After local US-directed anesthesia is performed, we shift the view in short axis and follow the needle by tilting the probe until we see a proper arterial tenting at 12 o’clock at the intended site of puncture, aiming to have the needle oriented at 45° degrees to the skin. In all the cases we have analyzed, we made a pre-closure with two suture-based vascular closure devices (VSCs).

### 2.3. Statistical Analysis

Numerical variables were reported as mean ± DS, or median and interquartile range (IQR), and categorical variables as number (*n*) and percentage (%). Continuous variables were compared between the two groups (echo-guided vs. non-echo-guided) using the *t*-test or Wilcoxon rank sum, as appropriate. Univariate logistic models were applied to investigate the possible association between the incidence of intra-hospital complications and type of procedure (echo-guided or non-echo-guided). A multivariate logistic model was used to eliminate possible confounding factors adjusted for propensity score, created as the following factors: age, sex (M, F), hypertension, diabetes mellitus, peripheral vascular disease, coronary injuries at the time of TAVI, NHYA (1, 2, 3, 4), BMI, EuroSCORE I, and EF (%). Considering the low number of complications, we also performed the logistic model for the incidence of the combined event. This variable was defined as the occurrence of at least one of the following complications: vascular complication access related, VARC-3 mortality, or bleeding sec VARC-3. All tests were 2-coded, and a *p*-value < 0.05 was considered sufficient for statistical significance. All analyses were performed with SAS Statistical Package v. 9.4 software (SAS Institute Inc., Cary, NC, USA).

## 3. Results

### 3.1. Baseline Characteristics

The baseline characteristics of the population are listed in Table 1. Body mass index (BMI) and body surface area (BSA) were higher in group B (mean and standard deviation 26.28 ± 4.4 vs. 27.29 ± 5, *p*-value = 0.01 and mean and standard deviation 1.8 ± 0.2 vs. 1.83 ± 0.2, *p*-value = 0.03, respectively). Interestingly, although this group had a higher BMI and BSA, thus implying a more uncomfortable and complex femoral access, they did not experience higher access-related complications, witnessing a favorable aid of the ultrasound tool.

The EUROSCORE I log. was found to be significantly higher in the non-echo-guided group (11.4 vs. 8.1, *p*-value < 0.0001). We used this old risk factor calculator, as it was the most adopted at the early stage of the study.

All other cardiovascular risk factors, such as diabetes mellitus, high blood pressure, chronic obstructive pulmonary disease (COPD), and a history of coronary artery disease, showed no significant difference between the two groups.

The only preoperative risk factor that was different between the two groups was the baseline past neurologic dysfunction.

In Table 2, the patients’ medical histories and pre-operative conditions are shown, with particular attention to their cardiovascular state and the implanted THV. We found differences in coronary artery disease with previous coronary artery bypass grafting (CABG) or percutaneous coronary interventions (PCI) and both mitral and aortic insufficiency that were more frequent in the blind group (group A). Both latter valve diseases were stratified by severity; we found these variables to not have any impact on our study objective.

All other preoperative clinical conditions showed no differences between the two groups.

Regarding the THV, we used S3 and S3U iterations. The diameters implanted were as follows: 23 mm (41.7% in group A, 35.3% in group B), 26 mm (45.7% in group A, 45.1% in group B), and 29 mm (12.6% in group A, 19.7% in group B).

In the blind group, the prosthesis models used were the S3 for 66.1% and the S3U for 33.9%; in the echo-guided group, the S3 was used for 19% and the S3U for 81%.

For the statistical purpose of greater homogeneity of the data, we conducted the analysis on 23 and 26 mm THV, requiring a 14 French (F) sheath. Thus, we excluded the THV requiring 16F sheath (i.e., 29 mm S3) because of the small sample of large annuli we have treated in the period of the study. Nevertheless, we saw a strong tendency to have no increase in vascular injuries using larger sheaths for this larger THV.

### 3.2. In-Hospital Outcomes

Table 3 depicts the intra-, peri, and postprocedural complications according to VARC-3 criteria [9]. The overall incidence of intraprocedural or periprocedural complications did not significantly differ between the echo-guided and non-echo-guided groups [30 (7.1%) cases in the non-echo-guided group and 15 (8.5%) in the echo-guided group]. Similarly, no statistically significant periprocedural differences were found between the two groups regarding overall and cardiovascular mortality, conversion to sternotomy, aortic dissection, prosthesis embolization, need for cardiac resuscitation or extra corporeal membrane oxygenation (ECMO), myocardial infarction, or major neurological events according to Valve Academic Research Consortium-3 (VARC-3) criteria.

However, in the non-echo-guided group, there was a clear increase in all bleeding events (life-threatening, major, and minor), with a *p*-value of 0.00001.

Considering overall vascular complications, there was no statistically significant difference between the two groups, with 36 events (8.6%) in group A and 13 (7.4%) events in group B. Even considering only access-related vascular complications, there was no significant difference between the two groups, although we observed a trend toward fewer access-related vascular complications in the echo-guided route.

Considering the low number of events (only 31 events in the non-echo-guided group and 11 in the echo-guided group), we analyzed the combined risk of periprocedural bleedings and access-related vascular complications and found a statistically significant difference between the two groups, with 73 (17.4%) combined events in group A and 15 (8.6%) events in group B, with a *p*-value = 0.005 (Table 4).

Finally, we have calculated the odds ratio of bleeding events and combined access-related complications (access-related vascular complications and bleeding events) and found a significantly higher risk in the non-ultrasound-guided group (for bleeding events, OR 0.13 with *p*-value 0.0007, and for combined access-related events, OR 0.45 with *p*-value 0.0069). To eliminate possible confounding factors, we set up a propensity-score analysis using baseline patient characteristics (Table 5). We found that both categories maintained statistical significance, with an adjusted OR for bleeding events of 0.13 (*p*-value = 0.002) and an adjusted OR for combined access-related complications of 0.44 (*p*-value = 0.01).

The associations also maintained statistical significance after adjustment for propensity score, with an adjusted OR for bleeding events of 0.13 (*p*-value = 0.002) and an adjusted OR for combined access-related complications of 0.44 (*p*-value = 0.01).

## 4. Discussion

The main findings of this study are as follows:

1. Ultrasound-guided femoral puncture resulted in fewer vascular-access and bleeding complications in percutaneous TF-TAVI.
2. Even in obese patients with unfavorable access anatomy, the benefit of the echo-directed puncture over the blind one is strong.
3. Although larger sheaths (16F) were excluded from the in-depth analysis, we nonetheless found a trend in those benefits, witnessing that larger holes are not per se related to worse vascular outcomes.

Access-related and bleeding events still carry relevant morbidity in TAVI patients, despite tremendous improvements in latest-generation THV delivery systems and increased operator experience [10]. Although in-depth computed tomography (CT) analysis is routinely pre-operatively performed with dedicated software, the arterial puncture site still poses challenges due to the relevant peripheral morbidity burden of the classic TAVI patient.

In our study (as for our daily practice), we have chosen the femoral puncture site according to the standard pre-operative CT features, focusing most on the absolute diameter of the vessel, tortuosity, calcium burden (anterior versus posterior wall), and femoral bifurcation height [11].

There was no specific bias in the selection of the puncture site between groups A and B.

Even if the femoral route has been established as the gold standard for TAVI procedures and the ultrasound-guided approach has started to gain widespread popularity in recent years, there is currently a lack of clinical guidelines recommending the use of the echo-guided approach [12]. However, multiple retrospective studies and registries have shown a safer profile when the access is achieved without a blind puncture [13].

Kotronias et al. [14] found that systematic routine use of ultrasound-guided access puncture was associated with a significant reduction in access-related vascular complications as well as the need for post-procedural blood transfusion compared to a fluoro-assisted access.

In our center, we started to systematically perform ultrasound-guided punctures in December 2019, and our retrospective review confirms the literature trend, showing fewer bleeding events in the echo-guided group (11.9% versus 1.7%).

As previously shown, we found a statistically relevant difference even after VARC-3 stratification, demonstrating that the echo-guided group had fewer life-threatening (2.9% vs. 0%), major (6% vs. 1.1%), and minor bleedings (3.1% vs. 0.6%). In our study, vascular complications were not statistically different, probably due to the low number of events in both groups. In group A, vascular complications were 36 (8.6%), and in group B, 13 (7.4%).

Third- and fourth-generation transcatheter heart valve systems present substantial improvements in delivery profile, diameters, and navigability. Despite this, the rate of access-related complications remains high. Much recent evidence suggests that vascular complications may be as high as 20%, with severe complications up to 9% [15].

A significant morbidity is associated with access-related complications, as these patients required longer hospital stays and had a higher need for blood transfusions.

All this also translates into prolonged in-hospital stays and higher overall procedural costs [16].

Even if, in our series, the vascular complications rate was not statistically lower in the ultrasound-guided group, if we consider only vascular complications related to the access site (such as pseudoaneurysms, hematomas, arteriovenous fistulas, dissection of the femoral or iliac artery, and local stenoses, thus excluding other vascular districts), the combined number of patients experiencing an access-related complication was significantly lower in the echo-guided group (17.4% versus 8.6%).

Obese patients (i.e., those having the body mass index (BMI) greater than 25) are a particularly challenging subset for percutaneous femoral access. As reported in Table 6, we have observed strong evidence favoring the ultrasound-guided access in the overweight subsets, with a significant reduction of both bleeding and vascular-access related complications in the adjusted and unadjusted population. This witnesses once again that a precise, anterior femoral wall away from calcifications puncture allows the commonly used VCDs to perform properly, reducing local complications and hemostasis time.

In our experience, the use of ultrasound-guided access has lowered the number of overall access-related problems, and, as it does not have a steep learning curve or significantly lengthen the duration of the procedure, its widespread adoption could significantly improve the clinical benefits. This favorable trend is still evident in the subgroup of obese patients, who classically pose challenges in the percutaneous approach due to the unfavorable anatomy.

## 5. Conclusions

Among the complications of the TAVI procedures, the most frequent are the access-related and bleeding ones. There is still no clear consensus in the literature on the role of ultrasound-guided puncture in reducing access-related complications, but an increasing number of studies and experiences demonstrate how this technique can contribute to the reduction of these undesirable events.

Our results also move in this direction, demonstrating how, in our series of patients, bleeding events—both combined and stratified by severity—and the overall number of access-related complications (access-related vascular complications plus bleedings) were significantly lower in the ultrasound-guided arterial puncture.

A widespread use of ultrasound could, along with a rigorous pre-operative screening of potential vascular risk factors such as small vessel diameter and/or high calcific burden, be determinant in further reducing the number of access-related TAVI complications.

We need further multicenter studies on larger patient samples to analyze the impact of the US-guided percutaneous puncture technique in reducing access-related complications in transfemoral TAVI and to affirm the role of this technique as a gold standard in clinical practice.

## 6. Limitations

The main limitation of this study is its single-center retrospective observational characteristic. Although the overall sample size was adequate to obtain statistically relevant results, the number of events, including vascular complications and bleedings, was small in the two groups, making a proper comparison difficult.

## Figures and Tables

**Table 1:** Baseline characteristics of the patients and cardiovascular risk factors.

Baseline Characteristics		Non-Echo (Group A) *n* = 421	Echo-Guided (Group B) *n* = 179	*p*-Value
Sex	Male	230 (54.6%)	94 (53.4%)	NS
	Female	191 (45.4%)	82 (46.6%)	
BMI (cm^2^), mean (DS)		26.28 ± 4.4	27.29 ± 5	0.015
BSA mq, mean (DS)		1.8 ± 0.2	1.83 ± 0.2	0.039
Diabetes mellitus, *n* (%)		94 (22.3%)	45 (25.6%)	NS
Hypertension, *n* (%)		333 (79.1%)	144 (81.8%)	NS
Neurologic disfunction *n* (%)		8 (1.9%)	11 (6.3%)	0.0055
COPD, *n* (%)		73 (17.3%)	22 (12.5%)	NS
History of coronary heart disease, *n* (%)		165 (39.2%)	65 (36.9%)	NS
EuroSCORE I log, median (Q1–Q3)		11.4 (8;17.3)	8.1 (4;13.9)	<0.0001
EuroSCORE II median (Q1–Q3)		35.9 (31.2;42.2)	31.4 (23.9;38.7)	<0.0001

BMI = body mass index; BSA = body surface area; COPD = chronic obstructive pulmonary disease, NS = not significant.

**Table 2:** Past medical history and pre-operative condition.

Medical History and Pre-Operative Conditions		Non-Echo (Group A) *n* = 421	Echo-Guided (Group B) *n* = 179	*p*-Value
Aortic insufficiency (mild, moderate, severe), *n* (%)	Mild	176 (41.8%)	59 (35.1%)	0.0008
	Moderate	72 (17.1%)	14 (8.3%)	
	Severe	15 (3.6%)	3 (1.8%)	
Mitral regurgitation (mild, moderate, severe), *n* (%)	Mild	231 (54.9%)	64 (38.1%)	<0.0001
	Moderate	70 (16.6%)	12 (7.1%)	
	Severe	9 (2.1%)	2 (1.2%)	
Indexed end-diastolic volume, median (Q1–Q3)		94.5(74.5;123)	54(46;71)	<0.0001
Coronary injuries at the time of TAVI, *n* (%)		96 (22.8%)	19 (10.8%)	0.00069
CABG or PCI for TAVI, *n* (%)		90 (21.4%)	13 (7.5%)	0.00005
Previous cardiac surgeries, *n* (%)		62 (14.7%)	19 (10.8%)	NS
Pre-operative MI (<90 giorni, >90 giorni), *n* (%)	<90	16 (3.8%)	6 (3.4%)	NS
	>90	38 (9%)	15 (8.5%)	
Pre-operative aortic valve max. gradient median (Q1–Q3)		71(61;85)	71(62;79.5)	NS
Pre-operative aortic valve med. gradient mean (DS)		44.35 ± 13.9	44.91 ± 11.6	NS
Pre-operative hemoglobin (g/dL), mean (DS)		12.56 ± 1.6	12.74 ± 1.9	NS
Pre-operative creatinine (mg/dL), median (Q1–Q3)		1(0.9;1.3)	1(0.8;1.3)	NS
Pre-operative critical state, *n* (%)		6 (1.4%)	2 (1.1%)	NS
Pre-operative haemodialysis, *n* (%)		4 (1%)	0 (0%)	NS
Prothesis model, *n* (%)	Sapien 3	279 (66.1%)	34 (19%)	<0.0001
	Sapien 3 Ultra	143 (33.9%)	145 (81%)	
Prosthesis diameter, *n* (%)	23	176 (41.7%)	61 (35.3%)	NS
	26	193 (45.7%)	78 (45.1%)	
	29	53 (12.6%)	34 (19.7%)	

TAVI = trancatheter aortic valve implantation; CABG = coronary artery bypass grafting; PCI: percutaneous coronary interventions; MI = myocardial infarction, NS = not significant.

**Table 3:** Intra-, peri-, and postprocedural complications according to VARC-3 criteria.

Complications	Non-echo (Group A) (*n*, %)	Echo (Group B) (*n*, %)	*p*-Value
Bleeding sec VARC-3	50 (11.9)	3 (1.7)	0.00001
Bleeding sec VARC-3 - Life threating - Major Minor - Minor			0.008
	13 (3.1)	1 (0.6)	
	25 (6)	2 (1.1)	
	12 (2.9)	0 (0)	
Cardiovascular mortality < 30 days	8 (1.9)	2 (1.1)	NS
Intra- or periprocedural complication	30 (7.1)	15 (8.5)	NS
Sternotomy conversion	2 (0.5)	0	NS
Aortic dissection	0	1 (0.6)	NS
Prosthetic embolization	0	1 (0.6)	NS
Atrial fibrillation new onset	24 (5.7)	11 (6.3)	NS
MI > 72 h	4 (1)	0	NS
ECMO implantation	4 (1)	0	NS
Need for cardiac massage	8 (1.9)	0	NS
Rupture and bleeding of the apex	4 (1)	0	NS
Stroke	13 (3.1)	4 (2.3)	NS
VARC-3 mortality	7 (1.7)	3 (1.7)	NS
Vascular complication	36 (8.6)	13 (7.4)	NS
Vascular complication access related	31 (7.4)	11 (6.1)	NS
Combined event	73 (17.4)	15 (8.6)	0.006

VARC = Valve Academic Research Consortium; MI = myocardial infarction; ECMO = extracorporeal membrane oxygenation, NS = not significant.

**Table 4:** Odds ratio adjusted for propensity score for variables with significant differences in the two study groups.

Event	OR	95% Cl		*p*-Value
Bleeding Complication Sec VARC-3				
Unadjusted	0.13	0.04	0.42	0.0007
Adjusted	0.158	0.05	0.52	0.0025
Combined event				
Unadjusted	0.45	0.25	0.80	0.0069
Adjusted	0.44	0.23	0.83	0.011

VARC = Valve Academy Research Consortium.

**Table 5:** Variables used for the propensity score analysis.

Variables Used for the Propensity Score Analysis
Sex (M, F)
Hypertension
Diabetes mellitus
Peripheral vascular disease
Coronary injuries at the time of TAVI
NHYA (1,2,3,4)
BMI
EuroSCORE II
EF (%)
Age

M = male, F = female, TAVI = transcatheter aortic valve implantation; NYHA = New York Heart Association; BMI = body mass index; EF = ejection fraction.

**Table 6:** Statistical analysis splitting the study population according to body mass index.

Event	OR	95% Cl		*p*-Value
Overweight (BMI ≥ 25)				
Bleeding complication sec VARC-3				
Unadjusted	0.139	0.032	0.594	0.0078
Adjusted	0.163	0.037	0.717	0.016
Combined event				
Unadjusted	0.357	0.161	0.789	0.011
Adjusted	0.341	0.142	0.817	0.016
Normal weight (BMI < 25)				
Bleeding complication sec VARC-3				
Unadjusted	0.114	0.015	0.865	0.036
Adjusted	0.146	0.019	1.126	NS
Combined event				
Unadjusted	0.616	0.257	1.477	NS
Adjusted	0.601	0.237	1.524	NS

BMI = body mass index, VARC-3 = Valve Academic Research Consortium 3, NS = not significant.

## Data Availability

The data presented in this study are available on request from the corresponding author (privacy).
